# Erosion

**Published:** 1892-03

**Authors:** W. S. Elliot

**Affiliations:** New York


### Erosion.

BY. W. S. ELLIOT, M.D., D.D.S., M.D.S., NEW YORK.

Read before the Ohio Dental Society, held at Columbus, December, 1891.

Papers and discussions upon this subject have as yet presented but little towards a satisfactory explanation of the phenomena. References thereto seem too much of a generalization and though it is conceded by most that the affection is of a chemical nature, yet in the concessions no chemical reactions are given, consequently our insight remains dim and knowledge is not increased.

I propose to draw no conclusions other than what might reasonably be deduced from the premises noted.

I offer this paper only as a study, and if not convincing, it may lead the scientist to work out more complete demonstrations.

The glandular appartus of the mouth is part of the general digestive system whereby foods are reduced to the requirements of nutrition.

If we cannot know the ultimate features of the digestive act, we can know something of the process and the successive steps towards its consummation.

In the saliva we recognize a peculiar substance known as ptyaline, a nitrogenous principal coincident with the properties of living matter. To it is ascribed the function and power of transforming starch and cane-sugar and other glucosides into glucose or grape-sugar. Starch is a definite chemical compound represented by $C_6 H_{10}, O_5$.

Through analysis we find that such change is one of hydration, viz: the absorption of a molecule of water, resulting in a body having new and distinctive physical properties and capabilities, known as glucose or grape-sugar. The reaction is here shown:

$$\begin{array}{l} C_6 H_{10} O_5\text{, starch,} \\ \quad H_2 O\text{, water,} \\ \hline C_6 H_{12} O_6\text{, glucose;} \end{array}$$

and we further find that under the influence of progressive, or perhaps perverted energy, this molecular mass is split into two corresponding parts, and with this division is presented still other properties which fall under the nomenation of lactic acid: $2C_3 H_6 O_3$.

It is to be accepted that the energy which disposes to this transformation resides in the substance of the ptyaline. The change would seem to lie strictly within the range of chemism, but it is impossible to define this limitation. We know nothing of the ultimate features of the vital force and but little of its componency; still, its power is noted and also the blending of the forces in the final results. We therefore style these changes a chemico-vital act.

An elaborate analysis of the ptyaline discloses certain pyoid bodies having a likeness to embryonal corpuscles or to leucocytes, and possess like them amœboid movements. These are known as Leeuwenhock's globules. According to Rouget the more numerous are these bodies the more augmented is the production of sugar in the saliva. This has appeared especially so in cases of mercurial salivation. This fact will prove suggestive to us in these investigations. If we now go back to the consideration of the antecedents of perverted salivation we will find excessive metamorphosis of the glandular elements through impressions of the nervous system from causes still more remote, and perhaps undefinable; but which, could we comprehend all, would lead us by successive steps to a positive understanding of the subject under consideration.

In the study, then, of erosion, we shall take cognizance of these known physiological and pathological conditions and inferentially, at least, deduce reasons for the existence of the affection.

It is well known that the direct application of grape-sugar, as of rasins, honey, etc., to the teeth frequently causes quite sharp pains, indicative of neural disturbance and enhanced sentiency; and it is also a known fact that the persistent contact will cause the enamel to be pitted and its integrity more or less destroyed. While the molecular disintegration is thus apparent we follow in our desires to comprehend its chemical aspects as well.

Having observed the reduction of starch to grape-sugar, then to lactic acid through a chemico-vital process of hydration, we reach the phase of the subject to which assent is generally given, namely: that the immediate cause of erosion is that of an acid saliva. But this statement is only a half truth in that the estimate is made as being altogether within the limits of chemical force. The acid condition is, as has been shown, an elaboration of vital as well as chemical influence, and as such is possessed of properties in which both qualifications are combined. A parallelism is that of stomachal digestion. Lactic and hydrochloric acids are here the products of the deliguium of the peptic glands associated with peptones in their career of chylification. Chemism, then, is not the only agency in the reduction and of course will not be so considered. Further, it has been the tendency to refer this acidity to the muciparous glands, but I think this reference is erroneous since the saliva is a complete production, and to it, as a whole, do we ascribe its functional capabilities. It is true that erosion is noticed more particularly upon the teeth in the immediate neighborhood of these glands, but there are special anatomical reasons why this should be so. Upon the incisors there is the additional agency of friction of the lips, and upon the molars that of the cheeks; and along the cervical aspect of the teeth where erosion is evident, there is retention of the saliva owing to the pocket-like character of the environment. These mechanical movements of the muscles are analogous to the churning movements of the stomach which assist in the liquification of the substances acted upon.

The reactions of cane-sugar are similar in kind to that of starch or glucose, though the disturbance may perhaps be less in degree. The change follows the same law of hydration, which is shown by the following:

$$\begin{array}{ll} C_{12}\ H_{22}\ O_{11}, & \text{cane-sugar,} \\ H_2\ O, & \text{water,} \\ \hline C_{12}\ H_{24}\ O_{12}, & \text{glucose,} \end{array}$$

or $2C_6\ H_{12}\ O_6$, glucose and levulose, and $2C_3\ H_6\ O_3$, lactic acid

So far, then we note the same results, but now for the action of the products of hydration upon the teeth. You will notice that the trend of my argument is away from the chemism as such, but including it in the general results. If, as before stated, there is perversion of the glandular secretions and an increase of Leeuwenhock's corpuscles with augmented production of glucose and acid, then we must realize a proportional enhancement of tooth waste; and if we estimate this waste as a strictly chemical one we must consider what are the elements concerned in the reduction. On the one hand is the acid, on the other the calcific constituents of the tooth. In this interchange the principal product can be none other than the lactate of lime. But when we estimate it from a chemico-vital standpoint we may deem the waste as more of a process of digestion, involving the necessary presence of the ferment, to which we have alluded, with the probable evolution of various complex bodies which remain undefinable.

In the empirical treatment of the affection preference is given to the use of chalk or calcium-carbonate, locally applied. Science indicates this as rational, since the selective energy is divested from the teeth to the free medicament where the affinities are fully satisfied. But this procedure contemplates only the effects of impaired function, erosion being a symptom only. An understanding of antecedent conditions becomes a necessary inquiry, and if full appreciation is given to the proportion herein stated, we must look to systemic agencies as influencing the pathogenic elaboration of the glandular products. But here comes bewilderment since the field is so broadened that direct statement seems impossible. I am not prepared, therefore, to give indications that would serve as a guide to a rational diagnosis. Prof. Peirce recognizes a gouty diathesis as coincident with the affection. My own observations have not confirmed this, nor indeed can I express any convictions that are positive or seemingly at all tangible.

In referring to statements made by Dr. W. H. Trueman, it appears that he advocates the chemico-vital theory. He says: "Solution is not necessarily a chemical process. The idea that this destructive agent must be an acid having an affinity with the lime salts of the tooth has little but tradition to support it. The little cap which we frequently see, mainly of enamel, all that remains of a baby molar, is sufficient evidence that there may be, and is formed in the oral cavity a true solvent of tooth tissue. I know this effect has been produced by a normal physiological process." He further suggests the idea of stomachic digestion as parallel to erosion, and excludes chemism as being only a minor agent. Prof. James Truman says, in contradiction to this view: "Erosion and abrasion are extremely simple—governed by a law of chemical action"; and further, that "erosion is the result unquestionably of chemical solution." These opposing statements do not help us in our investigations. With the clinical facts before us, and a recognition of the main features of oral function we are led to such conclusions as the inferences would naturally point.

It is suspected that a distinction is made between the process of absorption of the roots of the deciduous teeth and erosion of the permanent ones, in that one is considered a digestive process and the other an exclusively chemical one. It is not, however, so stated. Dr. Trueman believes that there is formed in the mouth a true solvent of tooth tissue. He makes no reference to the exfoliation of the infant teeth, but only and always to erosion, which Prof. Truman denominates, as before stated, a purely chemical act. In this review the conviction is towards the oneness of the seemingly varied causes for the solution of the tooth tissue. In each instance the same energies are working towards the same results; generally within the bounds of physiological requirement and sometimes upon the side of destruction and death. We would anticipate objections to the views here enunciated, and answer, that the reason why there is not more extended evidence of erosion when the career of the glandular elements is so definitely pronounced, is that the normal limitation is that of the production of glucose, which is the only condition capable of assimilation. If starch, cane-sugar, and other glucosides are not thus reduced they do not feed the tissues and are not capable of proper alimentation. It is the excessive transformation, under the more remote systemic influences, of the glucose to acid that constitutes the abnormality. A minor degree of acidity may not be incompatible with proper functioning. Indeed, it is possible that it is beneficial, as it appears to be stomachial digestion.

The solution of the roots of the deciduous teeth does not depend more upon the formation in the mouth of a true tooth solvent, than upon the digestive properties of the globular elements of the sulci, for these are endowed with the same properties as are the products of the salivary glands. All animal tissues—the blood, muscles and mucous membranes in general possess the property to a limited degree.

If undue acidity and erosion are dependent upon antecedent conditions, then the immunity therefrom is in proportion to the infrequency of the first cause, and if a gouty diathesis is coincident with the affection, the absence of erosion is accounted for by the rare occurrence of the predisposing conditions. Should we follow out to a further limit the process of fermentation in the mouth, it will be observed that lactic acid and calcium lactate are also prone to decomposition. Foul and ill conditioned mouths that arise from total absence of hygienic care testify to this continued transformation towards an extreme degree of putrefaction. The break up of the molecule of lactic acid is formulated thus:

$$\underbrace{2C_3\,H_6\,O_3}_{\text{Lactic acid.}} = \underbrace{C_4\,H_8\,O_2}_{\text{Butyric acid.}} \times \underbrace{2C\,O_2}_{\text{Carbon dioxide.}} \times \underbrace{H_4}_{\text{Hydrogen.}}.$$

The active agency here is a specialized organism described by bacteriologists as of the order of vibriones; but indeed in all these various modifications of the oral secretions there are ferments equally varied in their nature and order as are the products.

But let me modify my first issue; that in the study of erosion superficiality of attention has been given to the subject, I can claim no more, since there is so much that is unknown and perhaps unknowable, that that which is attained is meager enough, and if the really known transcends my feeble elucidation I will await the promulgations of the more erudite with impatient interest.

---

DISCUSSION.

*Dr. H. A. Smith.*—I saw a case of erosion the other day which was of special interest to me, and I will therefore briefly describe it. A patient presented himself at the clinic who had what I termed an erosion of the central incisors upon the labial faces a few lines below the termination of the enamel. One was a devitalized tooth, the other was normal; both were attacked by erosion precisely alike. There are two or three theories which have been advanced explanatory of this condition. One is that we have interstitial waste or a diminution (what we have been talking about to-day) of the nutrition of a tooth, and in consequence we have a breaking down or wasting away of the hard tissue upon the surface of the teeth. The case I have described disproves this theory. Erosion was still active upon a devitalized tooth. Nutrition being suspended, there could not have been interstitial waste. Another theory advanced is the tissues of the free margin of the gum secrete a fluid which either dissolves or digests the enamel and dentine, producing the characteristic appearance seen in erosion. Surfaces of teeth are frequently eroded quite a distance from the margin of the gum. Why is not the layer of enamel attacked next to the tissue which eliminated the solvent? The etiology of erosion is certainly very obscure. Perhaps the most rational explanation of the cause of erosion of the teeth, is, that the mucous membrane overlying the particular territory affected, secretes an acid which dissolves the lime salts. Whether this is true or not could be easily tested by applying freshly prepared blue litmus paper to the surface of mucous membrane supposed to secrete the acid.

*Dr. W. S. Elliot,* New York.—As regards the cutting edge of these teeth, there is no aberration except that which would be instituted through the epithelium of the upper jaw and face or lower portion of the tooth. But it shows the ordinary appearance of attrition. It is an attrition which is helped along by the same condition of saliva which produces the erosion. It would be impossible to separate the conditions entirely on the labial surface of the incisors of human teeth. We have an erosion with an attrition of the lips, which would produce abrasion as well as erosion. Upon the cervical portion of the tooth we have pockets which will hold the saliva *in situ* until it has produced its erosive effects upon the necks of teeth. That there should be a tooth isolated and giving the appearance of an erosion, it does not seem to me proper that it should be referred to any one particular gland. It is my conviction that the saliva, as a whole, is a complex substance that produces these results, but not by a chemical act, but by the physiological act of digestion, which depends entirely upon the mucous globules or peptones.

Prof. Peirce's statement, that he recognizes a faulty diathesis, is something I cannot appreciate, and in all instances where a gouty diathesis has been apparent in families, in some members of these families different effects have been produced.

I desire to lay stress upon the idea that frequently the crystals or fermentive bodies are probably instrumental in producing erosion. When it comes down to a purely chemical process, I do not believe it exists in the mouth any more than it exists in the human stomach. It is a physiological process rather than a chemical one.

*Dr. H. A. Smith.*—I have nothing special to say further. Dr. Elliot referred to the fact that in the case which I described there was no solvent action. He rather ascribed it to friction of the lower lip. It was of such a character that friction of the lip had no effect. It was eroded in a fine groove. The elevations were distinct on the enamel, so that the lip had no friction with it upon the plain surface. The Doctor's theory, and those we have seen elaborated, are very good as far as they go, but the main difficulty is the circumscribed territory which is affected, and if the saliva is the true cause of this condition, why is it so circumscribed ?

